# Becoming more oneself? Changes in personality following DBS treatment for psychiatric disorders: Experiences of OCD patients and general considerations

**DOI:** 10.1371/journal.pone.0175748

**Published:** 2017-04-20

**Authors:** Sanneke de Haan, Erik Rietveld, Martin Stokhof, Damiaan Denys

**Affiliations:** 1 The Berlin School of Mind and Brain, Humboldt-Universität zu Berlin, Berlin, Germany; 2 Faculty of Philosophy, Theology and Religious Studies, Radboud University Nijmegen, Nijmegen, The Netherlands; 3 Department of Psychiatry, Academic Medical Center, University of Amsterdam, Amsterdam, the Netherlands; 4 Amsterdam Brain and Cognition, University of Amsterdam, Amsterdam, the Netherlands; 5 Institute for Logic, Language and Computation, Department of Philosophy, University of Amsterdam, Amsterdam, the Netherlands; 6 The Netherlands Institute for Neuroscience, Royal Netherlands Academy of Arts and Sciences, Amsterdam, the Netherlands; Central Institute of Mental Health, GERMANY

## Abstract

Does DBS change a patient’s personality? This is one of the central questions in the debate on the ethics of treatment with Deep Brain Stimulation (DBS). At the moment, however, this important debate is hampered by the fact that there is relatively little data available concerning what patients actually experience following DBS treatment. There are a few qualitative studies with patients with Parkinson’s disease and Primary Dystonia and some case reports, but there has been no qualitative study yet with patients suffering from psychiatric disorders. In this paper, we present the experiences of 18 patients with Obsessive-Compulsive Disorder (OCD) who are undergoing treatment with DBS. We will also discuss the inherent difficulties of how to define and assess changes in personality, in particular for patients with psychiatric disorders. We end with a discussion of the data and how these shed new light on the conceptual debate about how to define personality.

## Introduction

One of the central debates in the ethics of treatment with Deep Brain Stimulation (DBS) concerns its effects on patients’ personality [1–10]. Does DBS change a person’s personality? This is a very important question for (future) patients, patients’ relatives, DBS researchers, and neuro-modulation techniques more generally. At the moment, however, there is relatively little data available about what patients actually experience following DBS treatment. There are a few qualitative studies into the general experiences of patients with Parkinson’s disease and Primary Dystonia—some of which also address changes in the self [11–16], a few case reports [3, 17, 18] and two recent studies that explicitly target personality changes after DBS with Parkinson patients [19, 20]. To our knowledge, however no qualitative study has yet been conducted on personality changes with patients suffering from psychiatric disorders who are undergoing treatment with DBS. Here, we present the experiences of Obsessive-Compulsive Disorder (OCD) patients who are being treated with DBS in the nucleus accumbens.

OCD patients suffer from either obsessions, or compulsions, or a combination of both.

Obsessions are unwanted, intrusive thoughts or images that repeatedly pop up, such as sexual or aggressive visions. They cause anxiety and distress, and people try to ignore or suppress them, or to neutralize them through some other thought or action [21]. These neutralizing thoughts or actions can easily become compulsions. Compulsions are repetitive acts, such as hand washing, ordering, checking, counting, or praying, that people feel driven to perform in order to prevent or reduce anxiety or distress or to prevent some dreaded event or situation—even though it is clear that these acts are either unrealistic or excessive [21]. OCD can be a highly disabling disease, with patients spending almost all of their waking time on their obsessions or compulsive behaviours. Patients’ insight into the irrationality of their behaviour even adds to their suffering [22]. The World Health Organisation lists OCD as one of the twenty most disabling diseases [23]. OCD affects approximately 2% of the general population [24]. About 50 to 60 percent of patients respond well to treatment with Cognitive Behavioural Therapy (CBT) and/or medication [23, 25]. About 10 percent of the patients however, do not respond to any of these treatments [25]. For these patients, DBS can be a treatment option.

We interviewed OCD patients who were being treated with DBS about the changes they experienced following treatment. In a previous paper, we described the overall changes that participants reported regarding themselves, their life world and social interactions, the characteristics of their way of interacting, and their existential stance [26]. The changes regarding themselves referred to how participants’ mood, emotions, self-confidence, and assertiveness changed following DBS treatment [26]. In this paper, we present the results of a different part of the study that specifically targeted *personality changes*: we asked whether participants considered themselves to be *changed as a person*. For it is one thing to feel less anxious or more self-confident, but it is quite another matter to in addition feel like one has become a different person.

Before we provide an overview of participants’ experiences, we will first discuss some preliminary considerations regarding the question of changes in personality. We then describe the methods and participants in our study. We end with a discussion of the data and how these data shed new light on the conceptual debate of what defines one’s personality.

## I. Preliminary considerations

### A. Different definitions of changes in personality, self, or personal identity

When considering changes in personality due to DBS treatment, we first need to clarify which changes amount to a change in ‘personality’. The concept of ‘personality’ is not so easy to define–and neither are the related concepts of ‘self’ and ‘personal identity’. Moreover, these terms are not identical in meaning and much can be said about their respective differences. In this paper, however, we will gloss over these differences and use these terms interchangeably. Various definitions have been called upon to account for the effects of DBS on personality, such as narrative, relational, and naturalistic ones. We will discuss the main proposals in turn.

In their 2008 paper, Synofzik and Schlaepfer [2] proposed a ‘naturalist’ notion of personality: ‘Personality is understood as the complexity of a system in which low-level sensory, motor, or vegetative states are important and are legitimate parts of it’ (p.1514). With this very liberal notion, they aim to avoid a dualism between ‘personal and non-personal brain modules’ (p.1514); proposing a gradual continuum between basic and more complex personal brain systems instead. Since Synofzik and Schlaepfer include sensory and motor processes as part of one’s personality, this means that on their definition DBS for movement disorders affects personality too: ‘based on this notion it would not make sense to ask *whether* a personality is affected by a certain neurotechnological intervention or not, but on *which level* and to *which extent* it is affected.’ (p.1514, first italics added, second italics by the authors). They go on to argue that the concept of personality is not useful for determining the ethical status of changes brought about by DBS. In the end the only ethically decisive question is whether the changes are regarded as good or bad by the patients themselves.

Synofzik and Schlaepfer’s definition implies that virtually *all* changes are changes in personality. The appeal of this position is that it seems to dissolve the problem. But there is still the intuition that some changes more fundamentally concern the person *qua personhood*. Moreover, while we agree that it certainly makes sense to include bodily (and not just cognitive) aspects into the concept of personality, we fear their definition runs the risk of making the notion so liberal as to be practically vacuous. For if everything belongs to someone’s personality it is no longer a notion that can be used to make meaningful distinctions. Apart from this theoretical concern, their ethical criterion faces some problems too. It is obvious that the patients’ own evaluation of the changes is of central importance. However, the effects of DBS may be such that they render the patients’ own perspective unreliable, such as in the case of (hypo)mania for instance. Besides, their proposal provides no criteria for *how* the patient (and others) could assess whether the changes are good or bad. Furthermore, as we will see, the division between good and bad, or desirable and unwanted effects, does not necessarily coincide with the distinction between authentic and inauthentic, which we here take to refer to the ‘feeling of being oneself’ versus feeling alienated or ‘not oneself’ [6]. That is, a change in personality can be judged as unwanted by patients, but still they may say that this is actually more who they really are–providing a powerful reason to embrace the changes anyway.

In her ‘*Philosophical reflections on narrative and Deep Brain Stimulation*’, Schechtman [10] offers a careful consideration of personality changes in Parkinson’s Disease (PD) after DBS. She argues that a narrative view on identity helps to describe the threats to identity that DBS seems to raise, and importantly, that it helps to find ways to avoid or mitigate these difficulties. According to Schechtman’s narrative view on identity ‘our selfhood is essentially tied not directly to defining traits, but to our ability to understand ourselves and others in narrative terms. We are selves–and construct identities–insofar as we experience and live our lives as narratives.’ (p.4-5). Following this view, a threat to one’s identity ‘stems from the disruption of the narrative flow of a life, and the resolution of that threat comes from repairing the narrative thread.’ (p.5). Schechtman proposes that acute changes brought about by DBS may feel like threats because of their sudden nature and ‘mechanical’ origin, but that taking a broader perspective could help patients to regain a feeling of agency regarding these changes. After all, the patients deliberately chose to embark on DBS treatment, and seen in that light these changes could be incorporated in their ongoing stories. When it comes to long-term changes, Schechtman suggests that it could be helpful to help patients and their relatives before the treatment to already construct a story about how their lives will continue after the treatment, where the story will help them to regard the changes as being part of one’s same ongoing self.

The big advantage of this view is that it is inherently dynamical and that changes are therefore not automatically regarded as threats to one’s identity. It can also nicely account for and explain the special difficulties of acute changes following DBS treatment. There are also some major drawbacks, however. First of all, the narrative view on identity is highly constructivist; it seems that as long as one can tell a good story about one’s identity, all is fine. To be sure, Schechtman [27] does propose two constraints that a story should meet in order to form a proper identity-constituting self-narrative: the articulation and the reality constraint. The *articulation constraint* holds that the narrator ‘should be able to articulate both the basic features of her history and life situation–the facts of her autobiography–and the way in which her life hangs together, providing explanations for why she has acted as she has and why things have unfolded as they have’ (p.82). The *reality constraint* states that a self-narrative should cohere with basic observational facts about the world. Taken together, these two constraints are still very liberal though: there are many stories to tell that cohere with basic observational facts about the world—even conflicting ones such as whether a specific change is a DBS side-effect or not. On the narrative view then ‘the important thing is that the change *be understood* in a way that makes it part of a coherent personal narrative, one that patients and their close associates *can see as*, overall, self-expressive and self-directed.’ (p.6, our italics). A less constructivist-prone critic may object that I can understand and interpret things in a myriad ways, but when it comes to my personal identity or personality, I would prefer that those changes *are* self-expressive, rather than merely being squeezable into a plausible-enough story that I can tell myself and others. If one is eager to avoid self-deception, to aim for a convincing story is simply not ambitious enough.

Moreover, Schechtman interprets the changes following DBS at the outset as *adjustment* difficulties, which can be reduced by changing one’s narrative in order to maintain one’s sense of personal identity despite these changes. But this suggestion ignores the possibility that DBS treatment could affect one’s personality directly: that the changes are not only problems with adjusting to the new situation [28]. Crucially, sometimes the changes should call for a re-adjustment of the DBS settings rather than a re-adjustment of the patients and/or their loved ones. If one only has malleable stories to refer to there is no ground on which one could ever argue that a change is in fact alien; that it is purely an unwanted side-effect of an electrical intervention–an effect that should be diminished as much as possible, rather than be adjusted to.

Another disadvantage is that the narrative view offers no foothold for the difficult cases in which patients and their loved ones disagree on whether the changes are positive or authentic. Nor does it seem to leave room for doubts about whether patients’ self-assessments can be trusted. The narrative view on identity offers no criteria for how to choose between competing narratives. On what grounds could one choose between the narrative: ‘This is a side-effect of the DBS’ and the narrative ‘This is who I actually am’?

Discussing the impact of DBS on personal identity in patients with PD, Baylis [5] takes up Schechtman’s notion of narrative identity, but stresses that narratives are always constructed by embodied selves in interaction with others, and in a particular socio-cultural context. She dubs this ‘relational identity’ and defines it as ‘a dynamic, socially, culturally, politically, and historically situated communicative activity (based in narrative and performance) that is informed by the interests, perspectives, and creative intentions of close and distant others’ (p.517). Following this relational take on identity she argues that DBS does not pose a threat to PD patients’ identity, for three reasons. First, because there is no such thing as a ‘true self’ anyway: ‘DBS for PD is not (and never could be) a threat to personal identity because personal identity is a dynamic concept’ (p.522). Second, in every person’s life major life events can occur that will change the person. These changes are part of rather than a threat to the person’s identity. Third, Baylis argues that it is the illness itself and the attached stigmatization that negatively affect one’s personal identity rather than the DBS treatment. She does however agree that in cases such as pathological gambling following DBS treatment [29, 30] the DBS poses a real threat to one’s identity. In general, DBS is a threat when it undermines one’s agency ‘to such an extent that the person is no longer able to meaningfully contribute to the authorship of her own life’ (p.525).

Baylis’ relational view on identity has the advantages that it is dynamic, and that it acknowledges the role of socio-cultural influences on identity, as well as the role of the body in shaping one’s identity. Her dismissal of the idea of an authentic self is a bit too quick though; after all, authenticity does not need to be understood in static terms. Of course major life events do not automatically constitute a threat to one’s personal identity, but DBS is not just any event. Given the prominent role of the brain in who we are, it makes sense to pay careful attention to what happens when it receives electrical stimulation. Furthermore, Baylis rightly points out that it is likely that the illness and stigmatization affect one’s identity, but it does not follow that DBS could not *also* affect one’s identity. Finally, while Baylis acknowledges that it is deeply problematic when DBS threatens one’s ability to make informed and rational choices, the relational view on identity she proposes cannot account for such cases. For like Schechtman’s narrative view, it offers too little ground on which to question someone’s self-narrative.

In their ‘*Deep Brain Stimulation and the search for identity*’, Witt and colleagues [1] propose a ‘Core-Periphery-Model’ of personal identity. They argue that not all changes in what people think and feel and how they act count as changes in their identity. Only when ‘a person’s core attitudes change, she changes’ (p.506). Core attitudes should be understood as attitudes with a ‘foundational function’ (p.506) which means that when they change, other more peripheral attitudes change along with them. An alteration of one’s personal identity thus entails ‘a profound paradigm shift in her cognitive or practical stance to the world’ (p.507). They stress that some aspects of our personal identity are not so much made by us, but rather given to us, such as our biological make up. We thus ‘discover ourselves at least as much as we create ourselves’ (p.506). Following this idea, they are critical of what they call ‘activity models’ of identity (such as Frankfurt’s [31] model), in which a central role is given to the person ordering her beliefs and desires; choosing which ones to identify with and which ones to denounce.

The Core-Periphery model has intuitive appeal: it makes sense that some attitudes and convictions are more important for who you are than others. However, as the authors themselves acknowledge, their model yet lacks clear criteria for when an attitude should be considered ‘foundational’. How many beliefs and desires need to change along with an attitude for it to be foundational? Another appealing feature is Witt and colleagues’ emphasis on the given and discovered aspects of personal identity. This stands in stark contrast to Schechtman’s [10, 27] and Baylis’ [5] more constructivist views. As we saw, these constructivist views face the problem on what grounds patients (and their loved ones) could decide whether a change fits their personal identity or rather poses a threat to their identity. Unfortunately, however, the Core-Periphery model does not offer an answer to this question either. It aims to elucidate which changes are changes in one’s identity, but it does not provide any criteria for judging whether these changes are authentic or inauthentic. For even changes in core attitudes can of course be either authentic or inauthentic–as their own example of someone losing their belief in God shows ([1], p. 506–7).

Recently, Dings and De Bruin [4] proposed to use Gallagher’s [32] ‘pattern theory of self’ to better understand the effects of DBS treatment. They point to the variety of conceptions of self and personal identity that now figure in the debate on DBS and rather than choosing between these different conceptions, they propose to embrace all of them as capturing different aspects of the self. Gallagher [32] offers a tentative list of 8 aspects: embodied, experiential, affective, intersubjective, psychological/cognitive, narrative, extended, and situated aspects of the self. These aspects are organized in different patterns and ‘a particular variation of such a pattern constitutes what we call a self’ ([4], p.6). Applied to the effects of DBS this allows for comparing patients’ pattern of aspects before and after treatment.

The main strength of this theory of the self is that it is very encompassing, aiming to take into account all possibly relevant aspects of the self. Its main weakness, however, is that it is just a list; a heaping of aspects, without an account of how they relate. There are no considerations on their potential ordering, hierarchy or structure—but in the case of psychiatric disorders and their treatment the relevant questions precisely pertain to this structure, to the relation between aspects of the self. Besides, the drawback of its encompassing character is that becomes unclear which changes are *not* changes of the self. Much like Synofzik and Schlaepfer’s [2] naturalist definition, the result of their deliberately ‘inflationary’ (p.6) account, is that it no longer has discriminatory power. Finally, the pattern theory of the self provides no criteria for how to determine whether changes are alien or authentic to patients’ selves.

Summing up, there are two main questions that an account of personality or personal identity should answer in order to be of use for evaluating the changes due to DBS treatment. First, an account should help to determine which changes amount to changes in personality or *personal identity*—as opposed to changes that do not affect the person qua person. And, second, it should provide suggestions for how to assess the *character* of these changes; that is, how can one determine whether a change entails becoming more or rather less oneself? As we have seen, this second need for criteria for assessing the authenticity of personality changes is not sufficiently met by any of the previous accounts we have discussed, even though this seems to be particularly relevant for the ethical status of these changes.

We do not want to propose another definition here. For our study, we did not adopt a specific definition, but just asked participants whether they noticed any differences in the way they felt, thought, or acted following DBS treatment, and whether they felt they had ‘changed as a person’ following DBS treatment. Nevertheless their answers do shed new light on the definition question, as will become clear in the discussion section.

### B. The specific role of DBS

A second difficulty in the debate is that it is not so straightforward to determine which effects are specific to the stimulation per se. Patients often still take psychopharmacological medication, albeit in reduced doses. Little is known about the possible interaction-effects between DBS and medication. Moreover, at our department, DBS treatment also standardly encompasses cognitive behavioural therapy (CBT), and it is this full package that impacts the patients. This is not to say that DBS alone does not already have an impact, see for instance the double blind on-off study [25], but it seems to be the combination with CBT that makes it all the more effective [26, 33]. Moreover, some changes may rather be the effect of recovery as such–independent of the way in which this recovery is attained [11, 12]. It could for instance be that any substantial reduction of anxiety would entail many of the changes mentioned by our participants. Furthermore, changes may also be secondary effects: for example, when patients profit enough from DBS treatment to return to work, this may in turn have a great positive impact on their social life, day-night structure, and the overall experience of leading a meaningful life. Finally, so called ‘placebo effects’ may also play a role: being part of an exceptional experimental study, being carefully monitored by a team of specialized therapists, and raising hope may all contribute to the effects of treatment. Several studies on DBS for OCS included a blind or double blind on-off phase, reporting diverging ‘placebo effects’ [25, 34–36]. A recent randomized controlled study [37] tested the placebo effects of DBS for patients suffering from treatment-resistant Major Depression (TRD). After the operation and a quick optimization of the settings, patients either received active tailored stimulation in the ventral capsule/ventral striatum, or merely sham stimulation. Remarkably, they found that after a 16 week period there was no significant difference in response rate between the two groups: 3 out of 15 were responders in the active group, versus 2 out of 14 in the control group. This suggests that the effects of treatment may include a larger placebo effect than previously assumed. Another recent randomized clinical trial on DBS for TRD patients however, did find a significant difference between sham and active DBS in 16 patients, and an overall response rate of 40% in 25 patients [38].

The speed of changes might play a special role though: once good settings have been found, the effects of DBS typically set in much quicker than in other therapies. The transition is thus more abrupt. This makes changes more visible, and leaves less time to adapt gradually. This is true also for partners and family. This speed moreover heightens the question whether changes are a side-effect of DBS. For, as Glannon [39] remarks, gradual changes are less likely to disrupt ‘the necessary degree of integration .. to retain identity’ than acute changes. Besides, as Schechtman (10) points out, insofar as the ‘change occurs immediately and via what at least seems to be a relatively straightforward causal chain (…) this .. makes it difficult to think about the changes as in any way a product of the patient’s own will or effort’.

### C. Wanted and unwanted effects

What makes matters even more complex is that in the case of OCD and other psychiatric disorders, patients in fact *want* to change the way they feel and think and behave [2, 7, 9, 40]. For psychiatric disorders precisely affect people in their very personhood: psychiatric disorders affect how and what you think, how you perceive the world, and what you feel and do. It is the aim of treatment to reverse these changes. DBS is in this respect of course no different from psychotherapy or pharmacological treatment. In the case of somatic disorders such as Parkinson’s disease, it seems more straightforward to dismiss all changes that concern the way the patients’ feel and think and behave as unwanted side-effects (although the situation is actually more complex here too, see: [11, 12, 41, 42]). By contrast, in the case of OCD and other psychiatric disorders some of such changes are actually sought for.

Even though some effects on the personality of psychiatric patients are intended, this does not mean that there cannot also be unintended side-effects on personality. How to distinguish the intended from the unintended effects? Note that this is not the same question as the *desirability* of the changes: the intended effects are obviously desired, but the unintended effects on personality might also be welcomed. Think for instance of OCD patients who felt very happy under specific DBS settings [3, 8]: this is a side-effect in the technical sense of the word, but the patient may still welcome the effect. A natural solution to demarcate intended from unintended effects would be to compare the ‘new’ personality traits with the personality of the person before the onset of the disorder. However, for psychiatric disorders with an early onset, such as OCD, it may be very hard to determine who one would have been without the disorder. If from childhood or adolescence on, OCD has influenced what you spend your life doing, what preoccupies you, the situations you avoid, your relationships, your social life, your professional life, etc., this is likely to leave a mark. And without a fully developed pre-OCD personality to compare to, it is difficult to determine whether DBS treatment has changed one’s personality or rather restored one’s ‘original’ self [7, 43]. For other psychiatric disorders with a later onset, the premorbid state may not be so univocal either. These patients too are likely to be affected by having this disorder and dealing with it. The same goes for PD patients. As Johansson and colleagues [7] remark, the general idea of restoring patients’ personality to their premorbid state is attractive but not very probable: ‘Most of the patients with MDD [Major Depressive Disorder] considered for DBS have lived with the disorder for years or even decades. Considering the severe impact of the disorder; the depression, as well as the treatment, is not likely to leave the patients unchanged.’ (p.2).

One of our participants, ‘Adrian’ (to maximize privacy we do not use the participants’ own names), said the following:

P14: That is also a difficult issue; I will just right away point to a very difficult issue on that matter [i.e. personality changes]… I have sometimes wondered how I would have been, how Adrian would have been if he had not had this anxiety and compulsive disorder in his youth? Well, I know one thing for sure, one thing I can say right away: I would have been a totally different person then. (…) I would have become a completely different person. Because from the very beginning, from the first years that you get something like that [OCD], the very first weeks that you get it, you will adjust your life to it. You try to survive, you don’t do anything else but trying to survive to be able to go on. You adjust your whole life, all your habits, and all the things around you to that. You select the people around you on that basis, you try to influence people, you try to achieve anything to be able to continue. And thus you become… Because I have had this anxiety and compulsive disorder for about 45, 50 years, you become a totally different person. So I had already become a different person because of the anxiety and the compulsions. And after the operation, I have often wondered: how would I have been without the anxiety and the compulsions?’

### D. Assessment of changes

This brings us to another difficulty, namely *how to assess* which differences following treatment with DBS are expressive of being oneself and which differences are not. Participants who used to avoid birthday parties, or would only sit quietly in a corner, now do most of the talking. Is this a suspect side-effect of DBS? Or do they rather follow a natural inclination that used to be overshadowed by their compulsions? How to decide? Participants also asked themselves this question and used different strategies to answer it (see section VI).

A common suggestion is that the evaluation of changes should be based on whether patients judge them to be positive or negative [2, 39, 41, 44]. This position has much intuitive appeal: of course patients’ own judgment of the desirability of the changes is highly relevant. But even though patients’ own assessment is of central importance, it cannot be the whole story. First of all, this position cannot account for the difference between considering a change positive or negative and considering a change authentic or alien. It may be that ‘negative’ changes in behaviour may still be experienced as expressive of one’s authentic self, which could be a reason to endorse them, while ‘positive’ changes could be experienced as alien, which could be a reason to reject them.

A second problem concerns the *reliability* of patients’ self-assessments. It may be that a patient’s assessment is itself influenced by the DBS [9, 39, 45]. If DBS increases one’s impulsivity and decreases one’s propensity to engage in reflection, it is likely that the reliability of one’s self-reflection and self-assessment will also be affected. There are indications that DBS may influence patients’ decision-making; rendering them more impulsive [46, 47], which might explain cases of pathological gambling after DBS [29, 30]. Moreover, a recent study with 40 PD patients found that patients had become more impulsive, more self-centered, less persistent and less conscious three months after surgery [20]. Relatives also reported that patients showed a ‘lack of premeditation’: patients were considered to be ‘less thoughtful, more impulsive, and likely to act on the spur of the moment without regarding the consequences’ ([20], p.4). The patients themselves did not report such changes. The authors suggest that patients may have a ‘reduced level of self-perception in behavioural change’ following DBS treatment ([20], p.5). In our previous study several participants reported that they had in general become more careless [26]. The fact that they also did not worry so much about the changes they experienced, could be interpreted as a corollary of that general attitude—especially since their partners sometimes did worry about these changes. By analogy, someone who is under the influence of alcohol is unlikely to see their state as problematic so long as they are drunk.

Furthermore, a familiar side-effect of DBS for OCD is that it may induce stimulation-related (hypo)manic phases [25, 35, 36, 48–52]. In cases of such side-effects, patients may be very happy with these changes, but their account is obviously distorted. It could, however, also be that patients themselves over time reject their manic state as being ‘unrealistically good’ [3]. Whether or not this occurs is an empirical question [53]. At any rate, one should be careful not to dismiss patients’ experiences by unnecessarily pathologizing them [8].

Another proposal is that the criterion should be whether or not patients *identify* with the changes [8–10, 27]. This position circumvents the distinction between the desirability and the authenticity of the changes, but it too cannot account for possible problems with the reliability of patients’ self-assessments.

Given these reliability worries, it might be helpful to supplement patients’ self-assessments with the assessment of the changes by partners, family members, or good friends. Previous research indicates that in general changes—both for better and for worse—may be more readily noticed by close relatives [13, 20, 26]. The assessments of relatives are not decisive either, though, for they may not have known the patient without OCD. In addition, the changes may (negatively) affect their relationship with the patient, which might also influence their evaluation [43, 54].

In clinical practice, it will be the therapist who faces the difficult task of weighing these various perspectives and estimate their respective reliability. After all, it is the therapist who is responsible for offering optimal treatment and minimizing side-effects–including the effects of treatment on how patients behave towards others. As Müller and colleagues [55] point out, this latter social aspect is a ‘blind spot’ in many ethical discussions.

Summing up, we’ve identified the following four central questions concerning the effect of DBS treatment on personality changes: (1) Which of the changes patients experience following treatment with DBS amount to a change in *personality*? (2) Are the changes the patient experiences *due to DBS* specifically? And (3) how to *evaluate* these changes? Do the changes pose a threat to someone’s identity, or are they rather expressions of someone’s ‘true’ identity? (4) By *what means* could that be assessed?

## II. Methods & participants

The participants were interviewed by the first author, using an in-depth, semi-structured qualitative interview method. Eighteen people participated in the study; 10 women and 8 men, ranging in age from 26 to 65 years. All participants met the inclusion criteria for DBS treatment at our hospital. Severe co-morbid personality disorders were an exclusion criterion, including Obsessive-Compulsive Personality Disorder (for the full list of inclusion and exclusion criteria see: [25]). The interviews took place 6 to 91 months after the DBS operation. Eleven participants were responders at the time of the interview. Most participants were interviewed at the psychiatric hospital, except for two participants (P7, P14) who were interviewed at least once at their own home. Generally, participants were interviewed alone—in two cases (P5, P17) the participant’s partner was present, but did not take part in the interview. The interviews were videotaped (in one case audiotaped (P15)) and transcribed verbatim. Participants were recruited personally by the first author, after they gave their permission to contact them via their therapists at the department. They received oral and written information, and confirmed their participation by signing an informed consent. They could at all times withdraw from the study. The study was approved by the medical ethics committee of the AMC (approval number: W11_113 # 11.17.1006).

We applied ‘purposeful sampling’, which means that we selected an as diverse as possible group of participants in order to get a broad overview of the various kinds of changes that people experienced. Sometimes therapists would recommend including specific patients because they had special experiences and/or were particularly good at expressing themselves verbally. The first author analysed the interviews through open coding; e.g. labelling fragments using mostly the participants’ own words (‘in vivo codes’), or sometimes referring to a theoretical concept (‘constructed codes’). MAXQDA 10 software was used for the analysis. To diminish personal bias, three interviews (P3, P4, and P14) were coded together with the second author. For a more elaborate description of the methods and participants of this study, see: [26].

We deliberately did not provide the participants with a particular definition of ‘personality’, ‘person’, or ‘personality change’. As the debate shows, there is no consensus even among professionals on how to best define ‘personality’ and what constitutes ‘personality changes’. The purpose of our study was to offer an inventory of patients’ actual experiences following DBS treatment. We therefore did not want to exclude any conceptions of personality or personality change beforehand, as we were precisely interested in the experiences that patients would come up with themselves. We thus aimed to use the most neutral wording and phrasing of personality changes, asking participants whether they noticed any differences in the way they felt, thought, or acted following DBS treatment, and whether they felt they had ‘changed as a person’ following DBS treatment–leaving it to the participants themselves to clarify what they meant when they reported such changes.

## III. Participants’ experiences

Six common themes emerged from the interviews: (i) I have not changed; (ii) I have become more myself; (iii) This isn’t me; (iv) I have to get used to myself; (v) I have to find out who I am without OCD, and (vi) How to determine whether changes reflect who I am. The first two themes are mutually exclusive: participants either felt that they had become more themselves (13 out of 18) or that they had not changed (4 out of 18). One participant felt that she had overall, become less herself (theme (iii)). The experience of theme (iii), ‘This isn’t me’, was however also shared by other participants who only had some *specific* alienating experiences, for instance regarding libido or assertiveness while their overall experience was that they had become more themselves. The last three themes were shared across participants—regardless of whether they felt they had not changed or felt that they had become more themselves.

We will outline these six themes, and illustrate them with quotes from the participants.

### (i) I have not changed as a person

Several participants (4) did not consider themselves changed as a person. One of them (a non-responder) unambiguously finds that he is not changed at all–and his wife and family do not consider him changed either:

P19: I do not at all feel… it [DBS treatment] works on my complaints and nothing else. So that is exactly right, actually. Apart from the fact that it doesn’t work good enough. (…) Yes, but I have not changed: my interests have not changed, my feelings have not changed. (…) And I also don’t feel that my wife says something like: ‘oh, I don’t recognize this’–nor my parents, or anybody else.’

Another participant (a responder) explains that *he* has not changed, but that *his life* has changed a lot since he profited from the DBS:

P4: ‘And of course, it is a very different kind of existence all of a sudden; it is very strange all of a sudden–you are also a bit alienated from the world, because you did not have anything to do with the world for twenty years. But that has got nothing to do with the DBS; just with the situation as such. No, I actually never noticed something that… Or, I heard sometimes that people start to think differently about things, get a different opinion, but I have never experienced something like that or anything.’

A third participant (a non-responder) finds himself not changed; only some of his character traits have gotten a bit stronger:

I: And do you feel that you have changed as a person?

P3: No, I don’t, actually. No, just that the traits that I had, have become a little stronger, so to say. That kind of thing. (…) I did not become a different person, no. No, it is really.. Yes, making a joke more often, or something like that; that it strengthens those things.

I: And are those things that you recognize for example from before, from before the compulsions started?

P3: Yes, yes, certainly! I used to be like that as a child: somewhat more lively and not always very nice, but sometimes yes. And yes, somewhat more jokes. Yes, and somewhat more interests, some more ideas, more creative also.

A fourth participant says she has not changed, but that her general level of anxiety is somewhat lower, which allows her to do things that she couldn’t do before, like going to a birthday party:

P22: Yes, it is just that the general level of anxiety is somewhat lower, which makes that you just a little easier.. Last Sunday, I had a birthday party of my sister in law, and yes, otherwise I would not have gone. And I still hated it.. Absolutely nothing.. Yes, everything is just a little easier.

### (ii) I have become more myself

The question ‘did you change as a person?’ proved to be ambiguous: the one participant may say ‘yes, totally’, and the other ‘no, not at all’–while in fact they mean the same thing. That is, the one may say: “Yes, I have become a different person: I have become who I actually am, without OCD; I am getting closer to my real self.” And the other may say: “No, I am not another person, all these changes reflect who I am without OCD.” We have grouped both of these answers under the header of ‘I have become more myself’. The majority of the participants (13 out of 18, i.e. 72%) report that they feel they are more themselves now, or are becoming more themselves. Now that the impact of OCD has been lessened, they can finally be who they really are, without their compulsions.

P20: ‘I felt more like doing things, looked forward to doing things. Yes, that heavy load that I carried, that got lighter, that got less. I needed less time to pause at things, and think about things, so it took less time. Well, I was.. yes really changed 360 degrees… What is this?! Oh… Yes, sure I still had those compulsions, they had not gone, but it was less. It was less burdensome, it weighed less heavy on me. I simply had room for other things too. (…) I think that, with regard to person, personality, character, that I am still the same, really. I do feel that I am indeed more Lea. First, I was Lea with the compulsions and then it was actually mostly the compulsions and behind that was Lea. Because the compulsions actually dominated everything, really, everything revolved around the compulsions. It was: the compulsions–and Lea, like that. And now it is actually Lea… yes, she has compulsions, but it is actually Lea who is at the foreground now. That’s how I feel. And of course, I still have my compulsions, but the compulsions used to rule my life. The compulsions were in charge, ruled over me. And now I am a bit in charge again over myself.’

P16: ‘Before my complaints, I also was somewhat more extrovert and because of the complaints I have completely changed and because of the DBS now my old self gets back a bit. That’s how you should see that. It is just that there are twenty years between the start of the problems and now, so that is quite a long time.’

P5: ‘I think I am coming back to my own self. If you are in a fixed pattern for decades and through such an operation you get back to your own self more, than that is a good thing.’

P10: ‘It [the new behaviour] belongs to me. Yes. But because of the compulsions, I think, I have not taken any space, or any time for it. And I have also become somewhat more sure of myself, yes. (…) Yes, I am certainly more myself, yes.

P13: ‘[I have become more] who I was. But who I was, that was, yes in fact the boy until the last class of the elementary school, right, because I can remember that very well. But I have to add: it is not just the DBS, but it is the DBS in combination with therapy. And for me that was EMDR [Eye Movement Desensitization and Reprocessing]. I am cleaning up my whole past with that.’

Some participants indicated that they had become a completely different person, in some extreme cases within several minutes after switching on the DBS. This participant experienced an extreme difference (see also Box 1):

P14: ‘And then, at a certain point, after a couple of minutes, someone says: ‘Adrian, do you notice that you have changed completely, the last few minutes, that you are completely very different?’. And I had not realized that until I started thinking about it. (…) And then I said: ‘Of course, you are right, I have changed completely.’ (…) I often just say; before the operation I was Adrian-1 and now I am Adrian-2. To just put it in a simple way. (…)

The last years, I have the idea that I am arriving at that natural Adrian, because I see some things, or someone else sees some things… like: ‘hey that is what my father used to do’–such things, you know. I think, so that is in the genes, that could be. That’s my idea about it. But there are inconceivably many things in which you change. Think only of the fact that I simply listen to different music. That alone is already quite something. I used to listen mainly to music from my youth; The Rolling Stones, The Beatles–I was a bit of a Rolling Stones fan, actually. I used to listen to that a lot, that kind of music and some Dutch music too… first records and then CDs. But then I had my operation and somehow I arrived at Johnny Cash.’

(See also a case study on this participant: [17])

**Box 1 - ‘Adrian’**

‘Adrian’ (P14)

Adrian was one of the few patients for whom the DBS was immediately effective, and also very effective. He estimates that he lost about 95% of compulsions; a great result. Adrian is very happy with the changes and now that he is retired he can finally live his life the way he wants to. He feels he has totally changed as a person: he is much more open and spontaneous and enjoys being able to now join his wife to birthday parties and dinners with friends. He thinks he has become more himself, that this would have been who he would have become if he had not developed OCD at the age of 13. His wife, however, is not so happy with the changes. That is to say: she is happy that he suffers less from OCD, but she finds he has changed a lot, to the extent that she feels: ‘This is not the man I married’. She reports that he often does socially inappropriate things, like talking to strangers at the street or in shops and that he does not pick up on signals when people are annoyed by him. When they are with friends he talks a lot about the latest DBS research, but does not ask them much about their lives. Since she often feels embarrassed about his behaviour, she feels less like going out with him. They tried to change the DBS settings, but at other settings the compulsions immediately returned, leading to an unfortunate dilemma between the pressure of the compulsions and the pressure on their relationship.

### (iii) This isn’t me

Other participants also felt they had changed, but regarded at least some of the changes as alien to them; as side-effects of the DBS. A familiar side-effect of the DBS is for example an increased libido [25, 56, 57]. Whereas some people experience this as a return to their normal sexuality, for others it feels alien and just ‘too much’.

P1: I also noticed with the DBS that it changes your libido, for example. But with me.. That has actually always been disturbed, always been minimally present, and if that changes, that is so unknown that you are not at all.. (…) It feels very extreme; that you think: everybody can notice what is happening with me.. (…) But then I think: jeez, how is that for other people, how do they cope with that, you know. (…) It is a direct consequence of [DBS], really a kind of side-effect. (…) It doesn’t belong to me, really.

P17: Yes, the libido, that that was.. Pfff… Well I did not like that at all. (…) No, that clearly didn’t fit with who I am. (…) Yes, that was so strange (…) I felt like having intercourse all the time (…) And of course I do want to have intercourse, but that doesn’t have to be every day, not at all. Once every two weeks is fine for me too. But I had this idea that I wanted to jump on him.. Very strange. (…) Yes, it was really too much; that really wasn’t me, you know; I really felt as if there was someone standing next to me who has this, you know. (…) Luckily I could discuss it with [my DBS-therapist] and we have found a good change in the settings, and yes, now it’s good: now it’s me again. (…)

Another common experience is that with DBS treatment people become more fierce, assertive, and sometimes even aggressive [26]. Many participants (9) feel that they express themselves more, and that this reflects their own selves, without the subduing effects of OCD. Some participants (3), however, felt that their increased assertiveness did not fit them. For instance, spontaneous reactions in which they stood up for themselves would surprise them and make them feel uncomfortable:

P10: ‘I used to be so insecure that I did not dare to say anything, and now I do say it. Sometimes a bit too much, I feel.’

Still, participants may characterize such unwanted changes as expressing who they really are. Thus the fact that they do not like these changes does not imply that they consider them to be alien to them. The same participant for instance says about her change in assertiveness:

P10: ‘..it belongs to me, yes, but because of the compulsions I have not taken up any space or time for that for years. And now I have become somewhat more sure, yes.’

Whereas for the other participants the alienating effects of DBS were limited to specific experiences only, one participant (a responder) regarded the changes in her behaviour as *overall* alien to her. She felt that DBS treatment had made her less herself.

P17: I now have a short fuse. (…) When I was very depressed, I also had a bit of a short fuse, but now it’s more that.. I cannot do so many things at once anymore. What I used to be able to do. (…) I thought it had to do with my personality stuff. But I am doing therapy again in the place where I live and that woman knows me from when I was 15, and I now turn 34, so she knows me for a very long time already, and she said: “That did not really belong to you before”. I said: “No, that’s right”. Yes, and then it got confirmed what I also felt myself. And that has been after the operation really.

### (iv) I have to get used to myself

Irrespective of whether participants regarded the changes as becoming more themselves, they frequently mentioned that they had to get used to themselves, their new lives, and their new behaviour. For instance, the participant quoted above, who did not consider himself to be changed as a person, still needs to get used to his new, spontaneous behaviour:

P4: ‘I really need to get used to myself too, actually. From always making up excuses, never being able to keep appointments… And then I just say: ‘Yes, sounds fun’–and then I think ‘Is that me?’ A bit strange, really, I need to get used to myself too.’

(see also Box 2)

**Box 2 - ‘Peter’**

‘Peter’ (P4)

With Peter, it took very long before the DBS was effective, but now that it is, it has changed his life completely. His life used to consist of only compulsions (fear of germs, counting compulsions), and even in his few hours of sleep he would still perform compulsive rituals in his dreams. But now he has finally the time and energy to actually live his life. He says he has not changed as a person; his convictions are still the same, but his life has changed tremendously. The way he spends his time has changed, and he now has the mental space to be interested in the world around him. Also, he has to get used to himself, to his own spontaneous reactions that often surprise him. After decades of being severely ill and having virtually no social life, he is now able to spend much more time with his family and to enjoy that time without hindrance from constant compulsions.

Another participant felt she had changed, and she did not really like these changes. She doubts whether her newly emerged assertive behaviour really fits with who she is. On the other hand, she thinks that her present reactions do reflect who she really is, but that she still has to get used to herself (see Box 3). In order to get a feel for the complexity and ambiguity of these matters, here follows a longer excerpt from the interview:

I: Have you noticed any other effects of DBS?

P21: Yes, I think sometimes–my mother has died shortly before the operation, and I think sometimes that if my mother would see me again, that she would not recognize me. Yes, bodily, she would, but… she would find me very much changed. And I also find that I have lost quite some friends after the operations, who feel like; well, ‘It’s not Janet anymore, actually.’ I do think that I have changed a lot.

I: Yes. And in which respects, do you think?

P21: Well, I think that I just, yes, stand up for myself more. And for some people, that is just not comfortable; the relationship [with them] just changes because of that…I: And those are things that before you might have ignored, things that you now express, or..?

P21: Well, before it would probably have bothered me also, but I would not have been so clear about it. I think I am just much clearer in giving my opinion on things. I think that has changed, yes.

I: Yes. And do you have the feeling.. it is a somewhat difficult question, but do you feel that in that respect you have become more yourself, or do you sometimes think ‘does this actually fit me, to stand up for myself in this way?’…

P21: No, I feel like: does this actually fit me, is this still me? I cannot really give a positive interpretation to it. I find… What I also have, and that also bothers me a lot, is that my interest in the people around me has decreased significantly. (…) It sounds very strange to say that about yourself (…) I used to be on top of everything and listening, and this and that, and trying to help people–and now I am more like: whatever, it will be alright. Yes, it sounds.. it’s not like I don’t want to help anybody anymore, but I really feel more like; I will wait and see if there’s not somebody else who might help. I don’t find I need to be the one to step in for everything.

I: Yes, and would this be an example that you think ‘does this really belong to me, or what actually belongs to me: being very helpful or the way I am now’?

P21: Well, I think I am more like I am now, but I am still not used to it.

[She tells about a conflict she has with an old friend of hers, as an example of how she does not let people get away with unfriendly remarks anymore.]

I: Yes, and before you would kind of let it pass..

P21: I think so, yes. The relationship, the preservation of the friendship, was more important to me than the way in which I felt about something. Whereas now, I feel more like, well, too bad. If I end up having no friends anymore, then there is nothing I can do about it.

**Box 3 - ‘Janet’**

‘Janet’ (P21)

For Janet too, the DBS has helped to get rid of a large part of her symptoms. Although she is very happy about this, she does not like all of the other changes that came with the DBS treatment. For instance she always liked to read, but now she cannot concentrate enough to read even a magazine, let alone a book. And she liked to paint, but she now feels she has somehow lost her creativity. In social relations too, is has not always been easy: she now speaks her mind more easily, and has lost friends in that way. She is also less interested in others, and does not really like that aspect of her ‘new self’. Still, she feels that this is who she actually is as a person, that it was the OCD that had made her adjust so much to others.

### (v) I have to find out who I am

An important inclusion criterion for DBS treatment is that the condition is chronic and has not improved despite treatment with all available drugs, and cognitive behavioural therapy. This means that for all participants, their lives in the previous years, sometimes even decades of years, have been seriously impaired. For those participants who have profited from the treatment, the relief of their previously omnipresent compulsions implies a huge change—including the amount of time they now have at their disposal. How to live this new life? The question of what they wanted to do with their lives had simply not come up before, as life had been a matter of day to day survival. With the positive effects of treatment, a new horizon of action possibilities or ‘affordances’ opened up qua work and social life (see figure 1 in [58, 59]. For a few younger participants this meant the possibility to start living the life that they had not dared to dream about before: going to university, or starting a family. For other participants, however, it also meant coming to terms with all the possibilities that had been lost because of the OCD. Given its often early onset, OCD affects people during formative years; impacting their schooling, relationships, work opportunities, and family life. After all those years, not all options are still open.

P20: ‘I am still very much.. because the compulsions actually disappeared, well, a small bit is still there, but compared to what it was.. I am now in such a phase.. I am searching for myself still; who am I? And that is just, yes, that fluctuates a lot. (…) I am wondering what I could still do now (…) Yes and also my personality; who am I and what is my opinion, those kind of things. And no is no. (…) Yes that kind of things, and to think more about yourself and not carry someone else’s load (…) I am actually somewhat more searching for myself; where I stand and where I want to stand.’ (…)

‘It is no longer only the compulsions that are at the foreground. I do really feel that I am more Lea now. That is also a bit unnerving, because; who is Lea? I have put her away for years and years, you know. Like I said; [there was] always the mask, and it was always the compulsions that preceded everything; it was always only about those compulsions. That was all my life consisted of really: compulsions. Yes, and now I am considering; I am doing this voluntary job and I am now taking my time to consider what do I actually want to do later, you know, do I want to work, or not, how am I going to do that? How am I going to spend the rest of my life? That is what Lea wants and not what the compulsion wants.’

P13: ‘I noticed for instance that in the last years before the operation, the news could hardly interest me. I did follow the news, but well, what did I care, right? Whether some politician had left or not. But now I notice that I do follow all of it and that it interests me a lot. And yes, so many things: this afternoon I am going to a museum with a colleague. Yes, those kinds of things. And I find that I can enjoy that so much–and all those things were gone [before the operation]. Yes, and movies; because I notice that I rent a lot of movies now. And yes, that I am beginning to find out which genre I like most and.. yes, that kind of things. (…) You are actually starting to find out who you really are, right? Because it was gone, yes. (…) Because of the compulsions and also because of the bullying. Because if you hear every day that you are a looser, an asshole.. Five years long. Yes, then you will start to believe in that, right?’

### (vi) How to determine what belongs to me?

The majority of the participants (12) had been thinking about the question of how to determine whether the changes that accompanied the DBS treatment were side-effects or rather reflections that they were becoming more themselves. Participants used roughly four different strategies or a combination of them. The most common (9) was to compare their present way of being with how they used to be before the onset of OCD. For some participants this was difficult, however, because of the early onset of their complaints. Several participants also referred to how others (family members, friends, a therapist) had known them, and whether they recognized the participants’ ‘old selves’ in their new behaviour with DBS. One participant (P14) compared his new behaviour to what his father used to do, and regarded the similarities as a sign that his current way of acting must reflect his real, OCD-less self. Another participant (P10) evaluated her changed behaviour in terms of how pleased she felt with it: if she felt good about it, she judged it as becoming more herself.

P18: ‘That was a good example; my oldest sister she noticed that too: she saw me at a few birthday parties, and then at some point she says, because she knows me the longest, she is six years older than I am (…). So she has experienced me from an early age. And she said at a certain point: I see the old Peter from the old days again; the cheerful Peter, the mischievous Peter, the funny Peter (…). She says: I see him again, I haven’t seen him for a very long time. I liked to hear that. So I was always already like that. She said: you were always a very cheerful and spontaneous child, sometimes annoyingly so, because you didn’t.. because you were almost wild.’

## IV. Discussion

### A. Becoming a different person?

One of the biggest worries in the debate on the ethics of neuromodulation techniques such as DBS, is that it might alter one’s personality. Given this centrality, it is important to note that ‘becoming a different person’ is an ambiguous characterisation. One might think of person A becoming a totally different person B, but one might also think of person A becoming a different, more open, expressive, or impulsive version of herself. What participants recounted in the interviews, rather falls under the latter interpretation. That is, there are changes, important changes, but these changes are not *random* or completely discontinuous. For instance, many participants report that they have regained interest in things–which is a huge change, but it does not mean that they are interested in completely *different* things than before. Participants generally do not get different hobbies or interests. Even an exceptional and extreme case like the participant quoted above who started enjoying different music [17], already liked to listen to music before.

Many participants indicate that they are generally more loose, and assertive, and express themselves more [26]. Some participants explain that they have not changed their views on things, but merely dare to say them out loud now. They may have enjoyed the same things, but now laugh out loud. Or they may have been annoyed by the same things, but only now dare to express their annoyance. What changes is their behaviour, their reaction to others; their way of interacting with the world. This is of course quite a change—and one that can have a huge impact too: for when you express your preferences, this will affect your interactions and the relationships with the people around you. On the other hand, this change in behaviour is not a change of one’s fundamental views, one’s values, or one’s outlook on life.

Based on the data of our study, we suggest that in answering the question of whether one has changed as a person, one could either refer foremost to one’s interactions with the world or rather refer to one’s values, convictions, and general outlook. Both how you (inter)act as well as your outlook on life play a constitutive role in who you are and what your life looks like. For most of our participants it seems that the ways they act and interact have changed considerably, while their convictions and views have remained the same. This distinction between definitions could nicely explain why some participants say that they have *not* become a different person, but rather are more themselves now, whereas others say that they *have* become a different person; namely more themselves. Participants who say that they have become more themselves rather than a different person, could refer to a definition of a ‘different person’ in terms of a changed outlook on life. Since this outlook has not changed, they do not consider themselves to have become a different person. Their behaviour may have changed considerably, but they feel they can now act more in line with how they want to act. Another terminological issue could be that since they feel they have become more *themselves* in how they act, they do not consider themselves to have become a *different* person.

On the other hand, participants who do say they have become a different person precisely refer to their changed behaviour and interactions; pointing out also that they often surprise the people around them. What supports this reading is that several participants who say that they have become ‘a different person’, also stress that they have not changed their opinions and outlook on the world, as to prevent precisely a confusion of what ‘being a different person’ entails.

These two different takes on ‘becoming a different person’ could also explain some seemingly contradictory answers of participants. For instance we saw that one participant (P4) both said: ‘I have not changed’, but also: ‘I have to get used to myself’–which would be strange if one had not changed at all. But here his claim that he has not changed refers to there being no changes in his opinions and outlook on life, while the need to get used to himself refers to his changed spontaneous behaviour.

Whether the reported changes amount to a change in personality or personal identity depends on which definition one adopts. From a clinical perspective, the usefulness of the concept of personality and personality change depends on whether it helps to uncover experiences of patients that are relevant for their well-being and that might otherwise be overlooked. Our study indicates that there are changes in the way in which participants feel, think and behave, and that these changes have an impact on their lives. Some of these changes are experienced as side-effects and call for an adjustment of the DBS settings.

Coming back to the definitions of personality changes that we discussed in section 1, from this clinical perspective, the definitions that regard *all* changes as changes of the self are not very helpful—such as the very liberal accounts of Synofzik and Schleapfer [2] and of Dings and De Bruin [4] But neither are the definitions that regard virtually *no* changes as changes of the self, such as the very flexible accounts of Schechtman [10, 27] and Baylis [5]. And lastly, the definition of Witt and colleagues [1] is not very helpful in the clinic either, as it focuses on a person’s core attitudes (beliefs and desires) as the central locus of concern, whereas our study shows that in general participants did not change their attitudes and beliefs towards things, but rather their way of acting and interacting.

But maybe a thorough theory of the self or personal identity is not even needed for this clinical purpose; what matters most is that the relevant questions are asked. What these questions should get on the table is whether or not the changes that patients experience are side-effects that call for an adjustment of the DBS settings, or that require support from the DBS team. For that the therapist needs to find out (a) whether patients feel the changes ‘fit’ them; whether they feel they have become more or rather less themselves; and (b) whether there is any conflict between patients and their loved ones on how to evaluate these changes. There may be no need for an elaborate theory on personality to pay attention to this issue in the treatment trajectory.

Note also that in our study, the only person who saw absolutely no changes at all, was a non-responder and in fact profited the least of DBS (see [26] for a more elaborate overview of our participants). This raises the question whether it is even possible for DBS treatment to improve the OCD symptoms without having any effect on the personality or personal identity of the patient. Maybe changes with respect to patients’ obsessions and compulsions cannot be achieved without also affecting their personality in some way.

### B. Assessing the authenticity of the changes

Another finding from our study is the relevance of the question of the authenticity of the changes: participants were particularly concerned with the question of whether they had become more or less themselves following DBS treatment. The relevance of authenticity in case of DBS treatment has been noted before [6, 7, 60, 61] and our findings confirm this relevance. Authenticity is, however, a very difficult issue, in general, and even more so in these special circumstances. For how to assess if one has gotten closer to one’s ‘real self’? Does it even make sense to speak of a ‘real self’? There is much to say in favour of understanding ‘the self’ in terms of a dynamical process rather than as a fixed entity. But there are limits to these dynamics too: not everything goes. Some effects of DBS definitely feel alien to patients, whereas other changes are clearly experienced as contributing to a person becoming more themselves. On what basis can one assess the authenticity of an experience?

A natural inclination may be to evaluate whether one *likes* the new behaviour, feelings, or thoughts as compared to how one behaved, felt and thought before DBS [2, 4, 44]. If one likes one’s new way of being, there does not seem to be a problem. This strategy can take us a long way, but it cannot be the sole principle. For coming closer to your ideal self is not identical to becoming more yourself [6, 62]. And as we saw, some participants disliked changes that they nevertheless judged as reflecting who they are without OCD.

A different answer comes from Schermer [8] who argues that whether changes are alien to oneself or not depends on whether one *identifies* with these changes. The idea, following Frankfurt’s [63] theory of personal identity, is that we have a multitude of beliefs and desires, but what makes us identify with those beliefs and desires is our second-order endorsement of them. It is this choice of endorsement that reflects who we are. The obvious difficulty of this position is that it is unclear *on what basis* we can make that endorsement. Higher order theories of the self run the risk of an infinite regress [64]: for the second-order identification with the first-order desires and beliefs would itself require a third-order endorsement which would again require a fourth-order endorsement and so on. Besides, as Witt and colleagues [1] also pointed out, this model assumes an *active choice*–against the strong intuition that at least part of who we are is given to us rather than a deliberative choice.

As we saw, Schechtman [10, 27] proposes that what fits with one’s personal identity is what fits in one’s self-narrative. This shifts the question from whether changes are authentic to whether one succeeds in incorporating these changes in one’s self-narrative. This implies that whether or not changes are conceived as alien cannot be evaluated directly following the surgery but will depend on the person’s subsequent process of appropriation. Although this approach is nicely pragmatic and flexible, it comes at the cost of offering no ground on which to evaluate the changes non-pragmatically. The narrative identity view could claim that this is not a problem, since on their constructivist account such an evaluation may not even be possible. The unfortunate consequence of this view, however, is that it invites clinicians and patients to accept changes as a given. Any problems are seen as the patient’s failure to adequately adapt to these changes–rather than considering the option that the changes might be side-effects, calling for a change in the DBS settings rather than in the attitude of the patient.

Could our study shed new light on this issue? As we saw, the participants in our study used various strategies to determine whether the changes fitted them or not: comparing their new experiences with how they were as a child, with how family members and others remembered them as children, comparing their new behaviour to that of other family members, and also evaluating whether they liked the changes or not. There may not be one definitive solution to determining the authenticity of certain changes, but our study at least suggests that it is important to discuss this question with patients and their relatives. Since the question of how to evaluate the authenticity of changes after treatment is common to psychiatry in general [65], it would make sense to further explore the strategies of other patients and see if they might be beneficial for DBS patients too.

### C. Alienation due to the device

A worry that sometimes comes up in the debate on DBS and selfhood, is that the implantation of the DBS device would lead to alienation [6, 66]. This concern is usually inspired by the qualitative study of Schüpbach and colleagues [11] on the effects of DBS on patients suffering from Parkinson’s disease where they found that 6 out of 29 patients (20%) reflected on the relation between the implanted device and their body, and that 3 (10%) of them had difficulties accepting the device in their bodies. For one of these three patients, the difficulties with the device disappeared after making an artwork of her chest X-ray (p.1813).

In our group of participants, however, there was no one who experienced such an alienation [28]. Participants tended not to think about the implanted device, except when there were technology-related problems such as a tension from the leads being too tight, or when there was leakage of current. But alienation proved to be not a theme at all. As one participant put it: the DBS had become a part of her, like someone with a new hip would forget that there was something artificial inside her. A qualitative study with 42 PD patients similarly found that ‘the device was not a big issue’ and that most patients seldom thought about it [16].

### D. Comparison with previous studies on personality changes

As far as we know, there have been no qualitative or quantitative studies on the effects of DBS on personality with psychiatric patients, only a few case reports [17, 47]. There have been a few qualitative studies into the general experiences of PD and primary dystonia patients following DBS treatment [11–16]. Two of those mention no experiences of personality changes at all [15, 16], whereas two other studies [13, 14] only shortly mention the effect that a changed body and bodily capacities may have on patients’ identity.

Recently, two studies have directly targeted the issue of personality changes in PD patients following DBS treatment: one quantitative [20], and one mixed-method study [19]. Pham and colleagues [20] used a selection of personality measurement scales to test 40 PD patients both before and three months after surgery. Their main findings were that patients showed an increased impulsivity after DBS, and a decline of ‘Persistence’ (maintaining behaviour despite fatigue or frustration) and of ‘Self-Transcendence’ (where less self-transcendent means ‘more self-centered and less conscious’ (p.4)). Furthermore, according to close relatives, patients also showed increased ‘Lack of Premeditation’ (‘the inability to think and reflect on the consequences prior to engaging in an act’ (p.3)). Interestingly, patients rated themselves as unchanged on this point. The authors suggest that this could mean that patients may have ‘a reduced level of self-perception regarding their behavioural changes’ (p.5). The study was limited to standardized scales only and did not include any qualitative interviews with either patients or relatives, so it remained unclear how patients and their relatives experienced and evaluated these changes.

Lewis and colleagues [19] used several personality scales and also interviewed 27 PD patients and their caregivers both before surgery and three months to one year after surgery. They found that 6 patients considered themselves changed (22,2%). Three patients had become more quiet, or more serious and less motivated. Of the remaining three patients, two patients report ‘more fun, more laughing’ and a ‘change to the positive’, and one reported a ‘different awareness of life’ (p.75). What these changes amount to is unfortunately not very elaborately presented in the paper. Interestingly, Lewis and colleagues [19] found that more caregivers than patients considered the patients changed (10; 43,5%). Here too, the information provided is sparse: 2 patients were described as being more quiet; 1 as being more depressed and sensitive; 2 as more selfish; 2 as overestimating themselves; 1 as ‘behaving like a teenager’; 1 as more open and talkative; and 1 ‘more aggressive, less even-tempered’. In addition, 21 patients reported mood changes, 12 of them regarded these as positive (57.1%), 9 as negative (42.9%). The authors point out that the reported changes are not picked up by the existing quantitative scales–but that they do have an important impact on patients’ lives and that of their relatives, even leading to endangering relationships and family life.

An unfortunate limitation of this study is the way in which the qualitative interviews were structured. Patients and caregivers first had to answer whether DBS had changed their/the patient’s personality, a question to which only a ‘yes’ or ‘no’ answer could be given. Apparently only the participants who answered this question affirmative where then further questioned on which changes they perceived. However, as we have seen from our participants, quite a few said that they had *not* changed as a person; there were important changes but these were interpreted as becoming more oneself. It could thus very well be that a more explorative design would have revealed more patients to experience personality changes–even if these fall within the bounds of the kind of person they consider themselves to be. Similarly, the changes in mood were only sorted in the binary positive-change versus negative-change, whereas it seems likely that there may be more ambiguous mood changes as well.

The first qualitative study with PD patients following DBS was conducted by Schüpbach and Agid and colleagues [11, 12]. Their important study directed attention to the fact that not all side-effects of DBS could be captured by the standard instruments. They repeatedly interviewed 29 PD patients to ‘qualitatively assess the impact of STN stimulation on the patients’ personal, marital, and socio-professional life’ (p.1812). The study shows the complex reality behind the medical recovery: for many patients there proved to be a gap between the ‘objective’ measures of response defining the success of treatment, and their own experiences and life world [12]. They report that patients became ‘more direct in their approach to others’, were more talkative, impatient, and irritable, and that they ‘expressed their opinions more freely’ (p.1814). This is remarkably in line with our own findings with OCD patients [26]. For our present topic, the most relevant finding however was that 19 out of 29 (66%) patients experienced ‘a feeling of strangeness and unfamiliarity with themselves after surgery’ (p.1813), which is illustrated by two quotes: ‘“I don’t feel like myself anymore”‘, and: ‘“I haven’t found myself again after the operation”‘ (p.1813).

This finding plays an important role in the debate on personality changes following DBS, and it seems to stand in stark contrast with the findings of our own study. How to explain this difference? First of all, our studies concern different patient groups, with DBS at different (but closely connected) sites in the brain. While OCD patients undergo DBS precisely because they want to change their thoughts, feelings, and behaviour, for PD patients the main goal is rather to restore their motor capacities. Still it is remarkable how alike our findings are with respect to some of the main personality changes that are reported. Both PD and OCD patients report being more direct in approaching others, being more irritable and impatient, and expressing their opinions more freely. In general, one might characterize these changes as becoming less inhibited or controlled [26]. In the case of OCD patients this is typically a welcome correction of an overly anxious stand in the world [67]. For PD patients however, this might more readily tip over to disinhibition.

Furthermore, the contrast may not be that strong; after all, like the PD patients our participants too reported that they found themselves changed, and that they had to get used to themselves. However, in our study most participants assessed these changes as becoming more themselves, whereas the study by Schüpbach et al [11] seems to refer to a negative experience, to becoming less themselves. In order to better compare our results, it would be interesting to know where these feelings of strangeness and unfamiliarity precisely refer to: to a changed outlook on life or to changes in behaviour and social interactions? Or do they refer primarily to the changes in motor abilities and the relation of participants to their body? Schüpbach and colleagues note that the feelings of strangeness ‘were associated with a clinically significant apathy in some patients’ ([11], p.1814). This did not seem to be the case with our participants (although some of them did report feeling tired). Schüpbach and colleagues also point out that many patients had become so used to their handicap that they initially did not dare to trust the success of treatment and their new, restored bodily abilities. Another qualitative study with PD patients by Haahr and colleagues [13] also found that some patients had difficulties to trust their own changed physical abilities. In a more general sense, Haahr and colleagues remark that patients’ relation to their changing body implies that ‘they had to achieve a new understanding of the self and repeatedly re-define the relation between self and body’ (p.1234). Maybe this latter aspect is less of an issue for OCD patients.

### E. Practical recommendations

What practical recommendations follow from this study? First, we recommend including semi-structured in-depth interviews as part of DBS research to gain better insight into patients’ post-surgical situation. Standard tests fail to capture central effects of treatment on patients’ experiences and lives [1, 11, 12, 19, 26, 68, 69]. More research into patients’ experience would provide information that could be used to better prepare patients beforehand of what might happen during DBS treatment, and also to better support them during the treatment trajectory. Witt and colleagues [1] argue for the development of a standard measurement of changes in personal identity, but, as they acknowledge, at this stage there is still too little known about the effects of DBS to develop a standard test. Qualitative research will be needed first to find out what would be the relevant questions to include and what type of answers one could expect to find. It will be a challenge to subsequently design instruments that can validly and reliably measure the often subtle personality-related changes that patients experience [41, 70]. Besides, relevant effects are not limited to changes in personality [26, 58].

A better understanding of patients’ own experiences is thus crucial. Contrary to a widely shared conviction, however, we propose that the patients’ own evaluation of the changes should not be the sole consideration. In particular, the possibility that DBS might affect patients’ competence and reflective capacities (as occurs in a manic state) should be taken into consideration. This requires weighing the perspectives of patients, patients’ loved ones, and the healthcare professionals involved. This brings us to a second recommendation: further research into the experiences of partners, family, or close friends of patients could possibly help to get a more adequate picture of the impact of DBS, including its social impact.

Clinical experience suggests that the specific settings of the DBS device may play a role in the effects on personality. In particular, it seems that higher voltages lead to an increase in impulsivity. It would be worthwhile to test this. And more in general, effects on personality could be one of the focus points in the search for better optimization of DBS settings.

Another interesting clinical observation is that the explicit recognition of positive changes could play an important role in the recovery process: acknowledging progress seems to be a boost for increasing that progress. Therapists play a crucial role in making these positive changes explicit. Maybe the explicit realisation of positive changes gives patients the trust that they can get rid of their obsessions and compulsions, and that improvement is possible after all—contrary to their often long history of fighting their symptoms in vain. The experience of success may motivate them. Patients, however, often do not notice the changes in their behaviour immediately. In our study we found that many participants only first noticed that they spent less time on their compulsions after therapists or other people had pointed that out to them [26]. This was also found in a qualitative study with patients with Primary Dystonia: ‘patients had to reflect themselves in the reactions of family and friends to actually believe in, and confirm the effect of DBS’ ([14], p.2104). One of them reported that ‘everyone else saw the improvement before I did. I think that was because I was afraid to admit that it happened.’ (p.2104). For our participants, the reasons may be somewhat different though. It seems that the new behaviour just felt so natural as to go unnoticed.

## V. Conclusion

There are changes in the way in which participants feel, think and behave, and these changes have an impact on their lives. Regardless of whether these changes fall under one’s particular definition of ‘personal identity’, ‘personality’, or ‘self’, patients are confronted with these changes and need to relate to and deal with them. From a clinical perspective, it is important to acknowledge the possibility that DBS can have side-effects on personality in order to recognize when such changes call for an adjustment of the DBS settings—rather than a re-adjustment of patients and/or their loved ones.

The majority of the participants (13 out of 18) in our study regarded the changes in how they feel, think, act and interact following DBS treatment as being part of their recovery from OCD. They felt they had become more themselves in comparison to their previous life that was dominated by their OCD. Still, a few participants felt that some specific changes were side-effects of the DBS. Four participants felt they had not significantly changed, and one participant felt less herself following DBS treatment. Interestingly, the changes that participants referred to concerned their way of (inter)acting, and expressing themselves, but not their convictions or (moral) outlook on life.

For the clinical DBS practice, the important question is not the theoretical question of which definition of personal identity to adopt and the ensuing discussion whether these changes amount to changes in patients’ identity. The important question is rather one that patients themselves struggle with, namely *how to assess* whether these changes are expressive of themselves or not. Patients’ own assessment is of course central. Their difficulty is to evaluate what does and does not belong to them: the early onset and long lasting OCD has affected them, which makes the evaluation also a question of who one would have been without OCD. The patients’ own evaluation cannot be blindly relied on, however, since DBS for OCD is known to potentially induce (hypo)mania; a condition which renders one’s judgement less reliable. Besides, there may be other effects of DBS on one’s way of evaluating, such as increased impulsivity. The assessment of the changes by partner, family or friends can be helpful to provide a complementary perspective. One of the difficulties here is that they may not have known the patient without OCD (especially partners and friends). Also, the changes may have affected their relationship with the patient, which may in turn affect their evaluation of these changes. In the end it is the therapist’s responsibility to ensure the optimal DBS settings and overall treatment of their patients. It is thus up to them to take all these perspectives into consideration and assess the reliability of both the patients’ assessment and that of their partners, family or friends.
